# Differential Gene Expression between African American and European American Colorectal Cancer Patients

**DOI:** 10.1371/journal.pone.0030168

**Published:** 2012-01-19

**Authors:** Biljana Jovov, Felix Araujo-Perez, Carlie S. Sigel, Jeran K. Stratford, Amber N. McCoy, Jen Jen Yeh, Temitope Keku

**Affiliations:** 1 Department of Medicine/Gastroenterology, University of North Carolina at Chapel Hill, Chapel Hill, North Carolina, United States of America; 2 Department of Pathology, University of North Carolina at Chapel Hill, Chapel Hill, North Carolina, United States of America; 3 Department of Pharmacology, University of North Carolina at Chapel Hill, Chapel Hill, North Carolina, United States of America; 4 Department of Surgery, University of North Carolina at Chapel Hill, Chapel Hill, North Carolina, United States of America; 5 Lineberger Comprehensive Cancer, University of North Carolina at Chapel Hill, Chapel Hill, North Carolina, United States of America; Howard University, United States of America

## Abstract

The incidence and mortality of colorectal cancer (CRC) is higher in African Americans (AAs) than other ethnic groups in the U. S., but reasons for the disparities are unknown. We performed gene expression profiling of sporadic CRCs from AAs vs. European Americans (EAs) to assess the contribution to CRC disparities. We evaluated the gene expression of 43 AA and 43 EA CRC tumors matched by stage and 40 matching normal colorectal tissues using the Agilent human whole genome 4x44K cDNA arrays. Gene and pathway analyses were performed using Significance Analysis of Microarrays (SAM), Ten-fold cross validation, and Ingenuity Pathway Analysis (IPA). SAM revealed that 95 genes were differentially expressed between AA and EA patients at a false discovery rate of ≤5%. Using IPA we determined that most prominent disease and pathway associations of differentially expressed genes were related to inflammation and immune response. Ten-fold cross validation demonstrated that following 10 genes can predict ethnicity with an accuracy of 94%: CRYBB2, PSPH, ADAL, VSIG10L, C17orf81, ANKRD36B, ZNF835, ARHGAP6, TRNT1 and WDR8. Expression of these 10 genes was validated by qRT-PCR in an independent test set of 28 patients (10 AA, 18 EA). Our results are the first to implicate differential gene expression in CRC racial disparities and indicate prominent difference in CRC inflammation between AA and EA patients. Differences in susceptibility to inflammation support the existence of distinct tumor microenvironments in these two patient populations.

## Introduction

Colorectal cancer (CRC) remains the most common gastrointestinal cancer in the United States, despite recent improvements in the diagnosis and treatment of the disease. The incidence and mortality rates of CRC for African Americans (AAs) are higher than in the U.S. general population [1], [2]. Many epidemiologic and genetic investigations have focused on AAs [3], [4], [5], [6] with the goal of deciphering the reasons for such disparities. Whereas one cannot discount the contribution of socioeconomic factors, such as a more advanced stage of disease at diagnosis in AAs, other biological factors also contribute to the progression of colon cancer [4]; [7]. However, a biological basis for the existence of a more aggressive CRC in African American patients remains to be further elucidated. Genomic instability is a crucial feature in tumor development and there are at least 3 distinct pathways in CRC pathogenesis: chromosomal instability (CIN), microsatellite instability (MSI), and CpG island methylator phenotype pathways (CIMP) [8], [9]. Any or all of these pathways may contribute to a more aggressive CRC biology in African Americans. Recent genome-wide association studies in CRC have shown not only strong evidence for common single nucleotide polymorphism (SNP) association in a number of genes and chromosomal regions, but also genetic heterogeneity in CRC association in AAs versus EAs [4], [10], [11], [12], [13]. Different incidence of MSI and different level of methylation for functionally very relevant genes were also reported as a possible factors in CRC racial disparities [8], [14], [15].

We hypothesized that the gene expression profiles of CRC in African-American and European-American patients may reveal biological differences between the two populations that could explain the more aggressive cancer phenotype in African-Americans. Thus, we performed genome-wide gene expression profiling in a large set of tumor samples that were matched for selected clinical variables. We analyzed our results on gene and pathway levels to identify key differences in tumor biology between African-American and European-American patients.

## Methods

### Patients

One hundred and fourteen tumors (86 included in original analysis and 28 for validation study) and 40 normal tissues from de-identified CRC patients were obtained from the Institutional Research Board (IRB) approved University of North Carolina (UNC) Tissue Procurement Facility after UNC School of Medicine IRB approval for this study. Written informed consent was obtained from all patients. All samples were collected between 1999 and 2008 at the time of operation and snap frozen in liquid nitrogen. Patients with known familial adenomatous polyposis and hereditary non-polyposis CRC were excluded. De-identified data including race, tumor, node and metastasis (TNM), grade or differentiation, margin status, and survival were available for the majority of patients.

### RNA Isolation and Microarray Hybridization

All RNA isolation and hybridization was performed on Agilent (Agilent Technologies, Santa Clara, CA) human whole genome 4X44 K DNA microarrays at UNC. RNA was extracted from macrodissected snap-frozen tumor samples using All prep Kits (Qiagen, Valencia, CA) and quantified using Nanodrop spectrophotometry (ThermoScientific, Wilmington, DE). RNA quality was assessed with the use of the Bioanalyzer 2100 (Agilent Technologies, Santa Clara, CA). RNA was selected for hybridization using RNA integrity number and by inspection of the 18S and 28S ribosomal RNA. Similar RNA quality was selected across samples. One microgram of RNA was used as a template for cDNA preparation prior to hybridization to Agilent 4X44 K whole human genome arrays. cDNA was labeled with Cy5-dUTP and a reference control (Stratagene; Catalog Number # 740000; Agilent Technologies, Santa Clara, CA; [16] was labeled with Cy3-dUTP using the Agilent low RNA input linear amplification kit and hybridized overnight at 65°C to Agilent 4X44 K whole human genome arrays. Arrays were washed and scanned using an Agilent scanner (Agilent Technologies, Santa Clara, CA).

All microarray data are in MIAME compliant form and raw and processed data has been deposited in the Gene Expression Omnibus (GEO); see http://www.ncbi.nlm.nih.gov/geo/, accession number: GSE28000.

### Microarray and statistical analysis

All array data were normalized using LOWESS normalization. Data were excluded for genes with poor spot quality or genes that did not have a mean intensity greater than 10 for one of the two channels (green and red) in at least 70% of the experiments. The log2 ratio of the mean red intensity over mean green intensity was calculated for each gene followed by LOWESS normalization [17]. Missing data were imputed using the k-nearest neighbors imputation (KNN) with k = 10 [18]. Genes that were significantly up- or down-regulated were identified using Significance Analysis of Microarrays (SAM) [19]. SAM assigns a score to each gene on the basis of a change in gene expression relative to the standard deviation of repeated measurements. For genes with scores greater than an adjustable threshold, SAM uses permutations of the repeated measurements to estimate the percentage of genes identified by chance – the false discovery rate (FDR). Analysis parameters (Delta) were set to result in FDR≤5%.

### Network and gene ontology analysis

Differentially expressed genes were investigated for network and gene functional interrelation by Ingenuity Pathways Analysis (IPA) software (Ingenuity Systems, www.ingenuity.com; [20]. IPA scans the set of input genes to identify networks by using Ingenuity Pathways Knowledge Base for interactions between identified ‘Focus Genes’, in this study, the differentially expressed genes between AA and EA and known and hypothetical interacting genes stored in the knowledge base in IPA software was used to generate a set of networks with a maximum network size of 35 genes/proteins. Networks are displayed graphically as genes/gene products (‘nodes’) and the biological relationships between the nodes (‘edges’). All edges are from canonical information stored in the Ingenuity Pathways Knowledge Base. In addition, IPA computes a score for each network according to the fit of the user's set of significant genes. The score indicates the likelihood of the Focus Genes in a network from Ingenuity's knowledge base being found together due to random chance. A score of 3, as the cutoff for identifying gene networks, indicates that there is only a 1/1000 chance that the focus genes shown in a network are due to random chance. Therefore, a score of 3 or higher indicates a 99.9% confidence level to exclude random chance.

### Ten-fold Cross Validation (Ten-f-CV)

Ten-f-CV analysis [21] was used to select smaller representative set of genes for validation study by qRT-PCR. Using Ten-f-CV analysis we identified 10 genes that can predict the ethnicity of the patient for whom the array was done with an error rate of 6%, suggesting that these 10 genes are representative of the entire gene list.

### Quantitative real-time PCR

Validation of microarray results was performed on 28 CRC patients (10 AA, and 18 EA). Ten differentially expressed genes (identified by SAM and selected using the Ten-f-CV method) were validated by qRT-PCR. The hydroxymethylbilane synthase (HMBS) gene served as the housekeeping gene [22]. qRT-PCR was performed in duplicates using SYBR Green Gene Expression Assays (Applied Biosystems, Forester City, CA), which include pre-optimized primer sets specific for the genes being validated [23]. The validated genes were: Crystalline, beta B2 (CRYBB2), phosphoserine phosphatase homologue (PSPH), Adenosine deaminase-like (ADAL), V-set and immunoglobulin domain containing 10 like (VSIG10L), Chromosome 17 open reading frame 81 (C17orf81), Ankyrin repeat domain 36B (ANKRD36B). Zinc finger protein 83 (ZNF83), Rho GTPase activating protein 6 (ARHGAP6), WD repeat domain 8 (WDR8), TRNA nucleotidyl transferase, CCA-adding, 1 (TRNT1), and HMBS. Data were collected using the ABI PRISM 7500 sequence detection system (Applied Biosystems, Forster City, CA). qRT-PCR data for each sample were normalized using expression of the housekeeping gene HMBS. Graphs were prepared from normalized data relative to HMBS. Statistical analysis of these data was performed with a two-sided *t*-test or with a two-sided Wilcoxon rank-sum test if the expression data did not follow normal distribution.

## Results

### Identification of differentially expressed genes between AA and EA CRC patients

Patient population characteristics for 43 AA and 43 EA patients were matched by TNM staging (**Table 1**). The two populations were similar in age, gender and tumor localization. Forty non-tumor colon tissues (13 AAs and 27 EAs) were used for genetic comparisons of normal colon gene expression between AAs and EAs.

**Table 1:** Clinical characteristics of the study population.

*Characteristics*	*Whites (n = 43)*		*Blacks (n = 43)*		*p-Value* *
	*No.*	*%*	*No.*	*%*	
Mean Age (y)	67	n/a	62.7	n/a	0.18**
Gender					
Male	19	44.19	25	58.14	
Female	24	55.81	18	41.86	0.28
Location					
Right	17	39.53	22	51.16	
Left	21	48.83	21	48.83	
Unknown	5	11.62	0	0.00	0.06
Tumor Stage					
1	4	9.30	4	9.30	
2	16	37.21	16	37.21	
3	8	18.60	8	18.60	
4	15	34.88	15	34.88	1.00

*p-values based on Fisher's exact test.

**p-value based on t-test.

**Figure 1:**
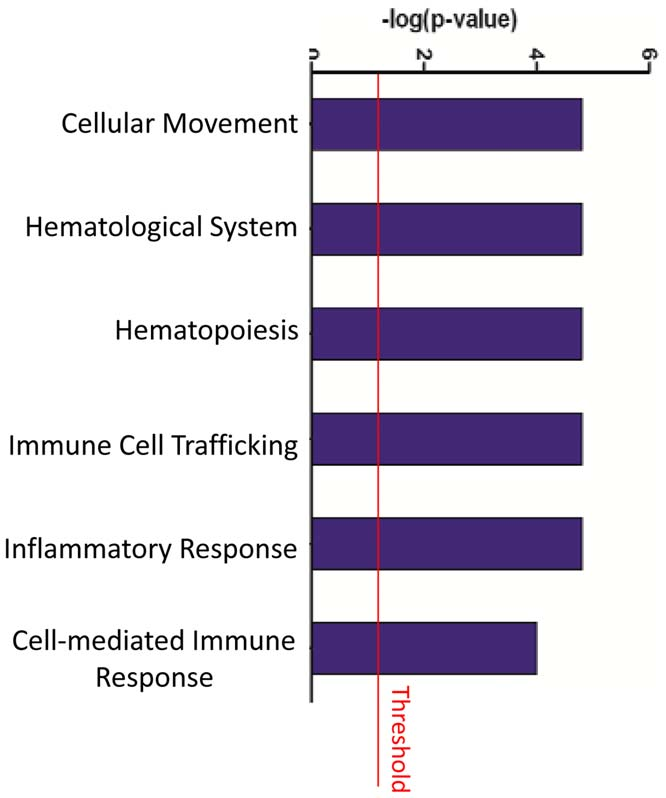
Ingenuity analysis of top pathways affected in differentially expressed genes between African Americans and European Americans. Y-axis is an inverse indication of p-value or significance. The Threshold line marks the p = 0.05. (Note that 3 of 6 top pathways shown are related to inflammation and immune response).

**Figure 2:**
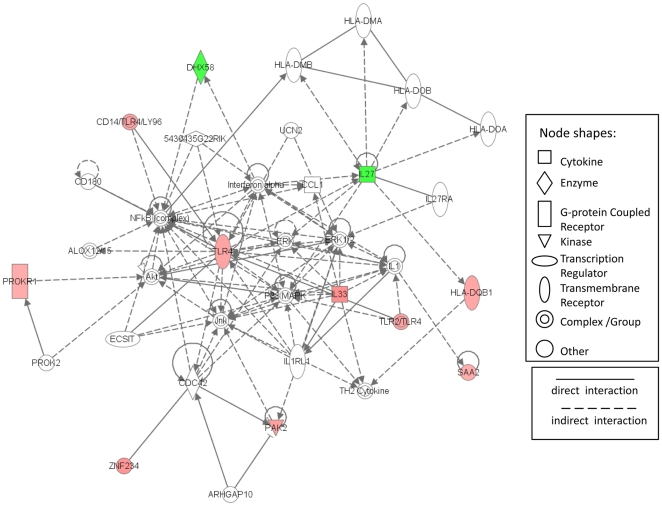
Gene network involved in “Inflammatory Response” generated by IPA for differentially expressed genes between African Americans and European Americans. Red symbols are assigned for up-regulated and green for down-regulated genes. Node shape corresponds to the functional role of molecules as shown in the legend. Direct or indirect interactions are shown by complete or dashed lines.

The comparison of gene expression profiles from AA and EA tumors using SAM revealed 95 gene transcripts to be differentially expressed between the two groups at FDRs of ≤5%. Fifty-eight genes were up regulated (**Table 2**) and 37 down regulated (**Table 3**) in tumor of AAs. We used Ingenuity Pathway Analysis to assess disease and pathway associations of these 95 genes that were differentially expressed in CRC tumors by race. The disease association analysis revealed associations of differentially expressed genes with genetic pathways that are linked to inflammatory response, hepatic system disease, developmental disorders, genetic disorders and neurologic disease (**Table S1**). The six top associated pathways for differentially expressed genes are shown in **Fig. 1**. Three of these six pathways are related to inflammatory and immune response. Differentially expressed genes in the five highest scoring networks are shown in **Table 4**. Top associated network functions for differently expressed genes were: 1) organismal injury and abnormalities, gene expression, cellular development 2) lipid metabolism, small molecule biochemistry, molecular transport 3) cellular assembly and organization, organ development, carbohydrate metabolism 4) antigen presentation and inflammatory response, cellular movement 5) behavior, digestive system development and function, endocrine system development and function. One of these networks (network 4; antigen presentation and inflammatory response) is graphically represented in **Fig. 2**. Seven of the nine genes in this network were up regulated in AA patients (HLA-DQB1, IL33, PAK2, PROKR1, SAA2, TLR4, ZNF234), and two genes were down regulated (DHX58, IL27).

**Table 2:** Up-regulated genes in colorectal tumors of African American patients.

No.	Gene ID	Gene Name	Gene Title	Chromosome Location	Fold Change
1	1415	CRYBB2	Crystallin,beta B2 Crystallin,betaB2	22q11.2-q12.1|22q11.23	3.10
2	5723	PSPH	Phosphoserinephosphatase	7p15.2-p15.1	3.38
3	116285	ACSM1	Acyl-CoAsynthetase medium-chain family member 1	16p12.3	2.47
4	23008	KLHDC10	Kelchdomain containing 10	7q32.2	1.48
5	90865	IL33	Interleukin33	9p24.1	3.08
6	6231	RPS26	Ribosomalprotein S26	12q13	1.63
7	401081	FLJ22763	Hypotheticalgene supported by AK026416	3q13.13	4.69
8	10780	ZNF234	Zincfinger protein 234	19q13.31	2.09
9	2863	GPR39	Gprotein-coupled receptor 39	2q21-q22	1.58
10	60439	TTTY2	Testis-specifictranscript, Y-linked 2 (non-protein coding)	Yp11.2	1.55
11	27012	KCNV1	Potassiumchannel, subfamily V, member 1	8q22.3-q24.1	2.58
12	55020	TTC38	Tetratricopeptiderepeat domain 38	22q13	1.67
13	256380	SCML4	Sexcomb on midleg-like 4 (Drosophila)	6q21	2.90
14	5062	PAK2	P21protein (Cdc42/Rac)-activated kinase 2	3q29	1.43
15	540	ATP7B	ATPase,Cu++ transporting, beta polypeptide	13q14.3	1.66
16	23562	CLDN14	Claudin14	21q22.3	2.35
17	10911	UTS2	Urotensin2	1p36	2.45
18	1416	CRYBB2P1	Crystallin,beta B2 pseudogene 1	22q11.2-q12.1	1.59
19	55282	LRRC36	Leucinerich repeat containing 36	16q22.1	2.45
20	348013	FAM70B	Familywith sequence similarity 70, member B	13q34	1.48
21	81839	VANGL1	Vang-like1 (van gogh, Drosophila)	1p11-p13.1	1.49
22	100190939	LOC100190939	HypotheticalLOC100190939	13q14.13	1.60
23	4552	MTRR	5-methyltetrahydrofolate-homocysteinemethyltransferase reductase	5p15.3-p15.2	1.74
24	348751	LOC348751	Hypotheticalprotein LOC348751	2q33.1	1.78
25	7099	TLR4	Toll-likereceptor 4	9q32-q33	1.95
26	5789	PTPRD	Proteintyrosine phosphatase, receptor type, D	9p23-p24.3	2.17
27	3043	HBB	Hemoglobin,beta	11p15.5	1.82
28	146456	TMED6	Transmembraneemp24 protein transport domain containing 6	16q22.1	2.61
29	253039	LOC253039	HypotheticalLOC253039	9q33.2	1.32
30	2037	EPB41L2	Erythrocytemembrane protein band 4.1-like 2	6q23	1.81
31	59352	LGR6	Leucine-richrepeat-containing G protein-coupled receptor 6	1q32.1	2.28
32	2689	GH2	Growthhormone 2	17q24.2	1.42
33	4886	NPY1R	NeuropeptideY receptor Y1	4q31.3-q32	4.03
34	283345	RPL13P5	Ribosomalprotein L13 pseudogene 5	12p13.31	1.99
35	3119	HLA-DQB1	Majorhistocompatibility complex, class II, DQ beta 1	6p21.3	1.55
36	140881	DEFB129	Defensin,beta 129	20p13	1.44
37	144568	A2ML1	Alpha-2-macroglobulin-like1	12p13.31	1.59
38	10783	NEK6	NIMA(never in mitosis gene a)-related kinase 6	9q33.3-q34.11	1.53
39	130399	ACVR1C	ActivinA receptor, type IC	2q24.1	2.32
40	27283	TINAG	Tubulointerstitialnephritis antigen	6p11.2-p12	1.97
41	116511	MAS1L	MAS1oncogene-like	6p21	1.47
42	8908	GYG2	Glycogenin2	Xp22.3	1.73
43	145447	ABHD12B	Abhydrolasedomain containing 12B	14q22.1	3.20
44	401577	LOC401577	Hypotheticalprotein LOC401577	Xp22.33	1.49
45	10887	PROKR1	Prokineticinreceptor 1	2p13.1	1.44
46	4889	NPY5R	NeuropeptideY receptor Y5	4q31-q32	1.50
47	327657	SERPINA9	Serpinpeptidase inhibitor, clade A (alpha-1 antiproteinase, antitrypsin), member 9		1.37
48	4860	NP	Nucleosidephosphorylase	14q13.1	1.47
49	1056	CEL	Carboxylester lipase (bile salt-stimulated lipase)	9q34.3	1.49
50	81796	SLCO5A1	Solutecarrier organic anion transporter family, member 5A1	8q13.3	1.34
51	6289	SAA2	Serumamyloid A2	11p15.1-p14	2.29
52	171558	PTCRA	PreT-cell antigen receptor alpha	6p21.3	1.31
53	8309	ACOX2	Acyl-CoenzymeA oxidase 2, branched chain	3p14.3	2.21
54	79857	FLJ13224	Hypotheticalprotein FLJ13224	12p11.21	1.28
55	9376	SLC22A8	Solutecarrier family 22 (organic anion transporter), member 8		1.33
56	7712	ZNF157	Zincfinger protein 157	Xp11.2	1.40
57	10	NAT2	N-acetyltransferase2 (arylamine N-acetyltransferase)	8p22	2.45
58	283422	C12orf36	Chromosome12 open reading frame 36	12p13.1	2.07

**Table 3:** Down-regulated genes in colorectal tumors of African American patients.

No.	Gene ID	Gene Name	Gene Title	Chromosome Location	Fold Change
1	57730	ANKRD36B	Ankyrin repeatdomain 36B	2q11.2	0.48
2	23587	C17orf81	Chromosome 17open reading frame 81	17p13.1	0.44
3	415	ARSE	Arylsulfatase E(chondrodysplasia punctata 1)	Xp22.3	0.21
4	395	ARHGAP6	Rho GTPaseactivating protein 6	Xp22.3	0.58
5	146547	PRSS36	Protease, serine,36	16p11.2	0.36
6	161823	ADAL	Adenosine deaminase-like	15q15.3	0.65
7	246778	IL27	Interleukin 27	—	0.36
8	644246	LOC644246	Hypothetical proteinLOC644246	17q21.31	0.42
9	79132	DHX58	DEXH (Asp-Glu-X-His)box polypeptide 58	17q21.2	0.54
10	51095	TRNT1	TRNA nucleotidyltransferase, CCA-adding, 1	3p25.1	0.60
11	55769	ZNF83	Zinc fingerprotein 83	19q13.3	0.65
12	64093	SMOC1	SPARC relatedmodular calcium binding 1	14q24.2	0.44
13	642946	LQK1	Hypothetical LOC642946	1q32.3	0.47
14	147645	VSIG10L	V-set andimmunoglobulin domain containing 10 like	19q13.41	0.65
15	26150	RIBC2	RIB43A domainwith coiled-coils 2	22q13.31	0.48
16	57531	HACE1	HECT domainand ankyrin repeat containing, E3 ubiquitin protein ligase		0.60
17	5303	PIN4	Protein (peptidylprolylcis/trans isomerase) NIMA-interacting, 4 (parvulin)	Xq13	0.62
18	8820	HESX1	HESX homeobox1	3p14.3	0.56
19	79609	C14orf138	Chromosome 14open reading frame 138	14q21.3	0.62
20	816	CAMK2B	Calcium/calmodulin-dependent proteinkinase II beta	22q12|7p14.3-p14.1	0.64
21	1953	MEGF6	Multiple EGF-like-domains6	1p36.3	0.55
22	79682	MLF1IP	MLF1 interactingprotein	4q35.1	0.51
23	51340	CRNKL1	Crooked neckpre-mRNA splicing factor-like 1 (Drosophila)	20p11.2	0.61
24	100287616	LOC100287616	Hypothetical proteinLOC100287616	15q24.1	0.54
25	201973	CCDC111	Coiled-coil domaincontaining 111	4q35.1	0.67
26	3712	IVD	Isovaleryl CoenzymeA dehydrogenase	15q14-q15	0.62
27	4942	OAT	Ornithine aminotransferase(gyrate atrophy)	10q26	0.60
28	57830	KRTAP5-8	Keratin associatedprotein 40306	11q13.4	0.50
29	388610	TRNP1	TMF1-regulated nuclearprotein 1	1p36.11	0.47
30	26152	ZNF337	Zinc fingerprotein 337	20p11.1	0.62
31	100287572	LOC100287572	Similar tohCG1996962	18q23	0.63
32	1427	CRYGS	Crystallin, gammaS	3q25-qter	0.65
33	4435	CITED1	Cbp/p300-interacting transactivator,with Glu/Asp-rich carboxy-terminal domain, 1	Xq13.1	0.55
34	339318	ZNF181	Zinc fingerprotein 181	19q13.11	0.69
35	49856	WDR8	WD repeatdomain 8	1p36.3	0.71
36	221322	C6orf170	Chromosome 6open reading frame 170	6q22.31	0.70
37	284018	C17orf58	Chromosome 17open reading frame 58	17q24.2	0.62

**Table 4:** Functional association of differentially expressed genes generated by IPA.

*ID*	*Focus Molecules in Network*	*Score*	*Focus Molecules*	*Top Functions*
1	ARSE, C6ORF170, CITED1, CRYBB2, CRYGS, EPB41L2, HBB (includes EG:3043), HESX1, HLA-DQB1, IVD, NAT2, OAT, PTPRD, RPS26, ZNF83	**28**	**15**	Organismal Injury and Abnormalities, Gene Expression, Cellular Development
2	ACVR1C, ATP7B, C17ORF81, GH2, GPR39, MLF1IP, NP, PIN4, PTCRA, SLC22A8, TRNT1, TTC38, VANGL1,WDR8 (includes EG:49856)	**26**	**14**	Lipid Metabolism, Small Molecule Biochemistry, Molecular Transport
3	ANKRD36B, CRNKL1, GYG2, KCNV1, KLHDC10, MEGF6, MTRR, NEK6, PSPH, RIBC2, ZNF337	**20**	**11**	Cellular Assembly and Organization, Organ Development, Carbohydrate Metabolism
4	DHX58, HLA-DQB1, IL27, IL33, PAK2, PROKR1, SAA2, TLR4, ZNF234	**15**	**9**	Antigen Presentation, Inflammatory Response, Cellular Movement
5	CAMK2B, CEL, NPY1R, NPY5R, SCML4, UTS2	**9**	**6**	Behavior, Digestive System Development and Function, Endocrine System Development and Function

We also performed SAM analysis using non-tumor colon tissues from AA and EA patients and did not see differential gene expression (data not shown), suggesting that the changes we identified are tumor microenvironment specific.

### Validation of microarray results

In order to select a representative group of genes for qRT-PCR validation of differentially expressed genes between AA and EA CRC patients, we performed a 10-fold cross validation analysis that resulted in the selection of following ten genes: CRYBB2, PSPH, ADAL, VSIG10L, C17orf81, ANKRD36B, ZNF835, ARHGAP6, TRNT1 and WDR8.

**Figure 3:**
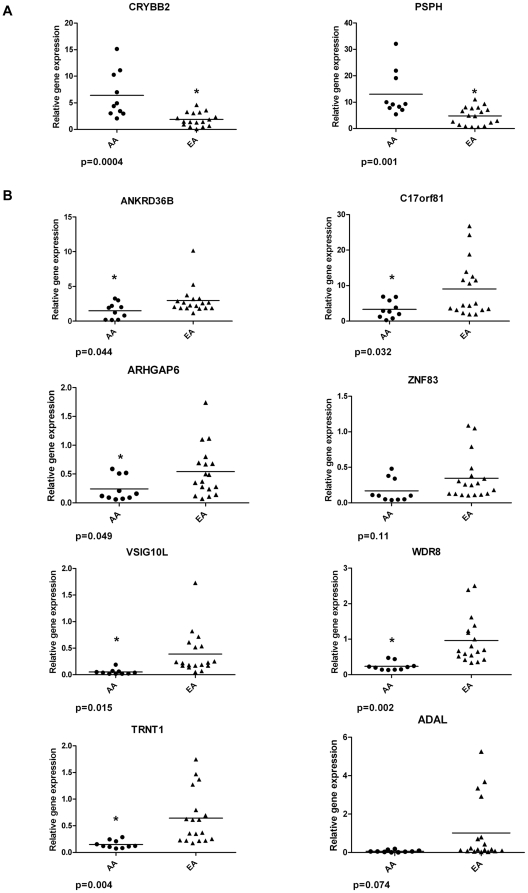
qRT-PCR validation analysis of expression of ten selected genes between African American and European American CRC patients. Panel A. Expression of two up-regulated genes in African American colorectal cancer patients: CRYBB2, PSPH. Panel B. Expression of eight down-regulated genes in African American colorectal cancer patients: ARHGAP6, VSIG10L, C17orf81, ZNF83, ANKRD36B, ADAL, WDE8 and TNRT1. Points are relative Ct values for the individual samples; horizontal lines are mean values for the sample set. The significantly differentially expressed genes (p<0.05) between African-American (n = 10) vs. European-American (n = 18) CRC tumors are labeled by a star. Graphs were prepared from normalized genes expression data relative to the housekeeping (HMBS) gene.

Expression of these ten genes was validated by qRT-PCR on an independent test set of 28 CRC patients (10 AA and 18 EA). The qRT-PCR results are shown in **Fig. 3**. Two of the 10 differentially expressed genes were up-regulated in AA vs. EA CRC patients; CRYBB2, p = 0.0004 and PHSP, p = 0.001 (**Fig. 3** **; Panel A.**). Eight were down-regulated in AA vs. EA CRC patients; VSIG10L, p = 0.015; C17orf81, p = 0.032; WDR8, p = 0.002; TRNT1, p = 0.004; ANKRD36B, p = 0.044; ARHGAP6, p = 0.049; ADAL, p = 0.074; ZNF83, p = 0.11; (**Fig. 3** **; Panel B.**). The direction of change (up or down regulation) for the qRT-PCR validated genes was in agreement with SAM results. Eight of ten genes in the qRT-PCR validation study reached a statistically significant level (p<0.05; CRYBB2, PSPH, C17orf81, ANKRD36B, VSIG10L, WDR8, TRNT1 and ARHGAP6).

## Discussion

The causes of the CRC disparity that exists between AA and EA patients remain to be fully elucidated. Although most of the research on this disparity has focused on socioeconomic factors, recent findings strongly support the role of genetic and biological factors. Genetic differences between AA and EA CRC patients were reported for SNP association, for incidence of MSI and level of gene methylation [4], [14], [15]. Any of these differences can result in differential gene expression between AA and EA CRC patients. In this study we analyzed the gene expression profiles of 86 tumors from 43 AA and 43 EA patients. Significant differences in the expression of genes related to immune response and inflammation within the tumor micro-environment were identified between these two groups. This interpretation was supported by both disease association and pathway analyses. Most of the immune-related genes had higher expression in tumors from African-American patients than in those from European-American patients. Although preliminary, these findings are novel and could have implications for cancer therapy. From the present study, we do not know why CRC from African-American patients would have a different immunologic profile than tumors from European-American patients. We hypothesize that the causes of these differences are multifactorial. Chronic inflammation is thought to be a causative factor in colorectal carcinogenesis [24], [25]. It was shown that an immune response signature in the liver of cancer patients predicts metastasis and recurrence of hepato-cellular carcinoma [26]. Thus, future studies should evaluate whether the immunologic profile of CRC in African-American patients is a predisposing factor for tumor progression and metastasis. Previous investigations identified a two-gene tumor signature (CRYBB2 and PSPHL) that accurately differentiated between African-American and European-American prostate cancer patients [27]. Those two genes were also differentially expressed between African-American and European American breast cancer patients [28] In this study we found up-regulation of CRYBB2 and PSPH gene in CRC of African-American patients. Mutations in the CRYBB2 gene are also responsible for familial cataract [29]. PSPHL is a homolog of PSPH. Interestingly, PSPH is located on chromosome 7p15.2, a chromosomal region known to have gain of function related to advanced tumor stage in non-small-cell lung adenocarcinoma [30]. It was shown that increased expression of PSPH in non-small-cell lung cancer corresponds to clinical response to treatment with erlotinib [31]. Thus, it is possible that higher expression of PSPH contributes to CRC susceptibility in AAs and that the levels of PSPH expression may be correlated with response to anti-EGFR treatment. These possibilities will have to be tested in future studies. Considering down-regulated genes in AAs we found lower expression of the C17orf81 gene. Down regulation of this gene was associated with colon cancer [32], suggesting that lower expression of this gene can contribute to more aggressive CRC in AAs. Other down regulated genes in AAs include: TRNT1 (involve in RNA processing; [33]); ARHGAP6 (promotes actin remodeling: [34]); WDR8 (facilitates formation of multiprotein complexes: [35]). Considering the cellular functions of these genes it is not hard to envision how their expression may influence aggressiveness of CRC.

Whole-genome gene expression analysis experiments can be prone to findings that are either unique to a selected patient population or are artificially created by the applied technology. To exclude the possibility of an artifact, two different approaches were used to cross validate our gene expression data. First, we compared our results of the differentially expressed genes between 86 tumors and 40 surrounding non-tumor tissues with those from a published meta-analysis of five CRC gene expression datasets in Oncomine [36], [37], [38], [39], [40]; **Table S2**. We found a very good agreement between our results and the results of the other 5 meta-analyses. Of the top 20 over-expressed genes in CRC tumors across the 5 other meta-analyses (Oncomine; https://www.oncomine.org), 15 were also found to be significantly up-regulated (FDR, <5%) in our study. Of the top 20 under-expressed genes in CRC tumors across the 5 other meta-analyses, 17 were significantly down-regulated (FDR, <5%) in our study.

Second, we validated the expression of ten key genes via qRT-PCR and confirmed differences in gene expression between CRCs of AAs and EAs for eight of them.

In conclusion, the gene expression profile of CRC corresponds to differences in tumor biology between African-American and European-American patients. The implications of these differences in disease aggressiveness and response to therapy should be evaluated in future studies.

## Supporting Information

Table S1 **Ingenuity Pathway Analysis of association of differentially expressed genes with top bio functions.**
(DOCX)

Table S2 **Comparison of top 40 differently expressed genes in five other CRC microarray studies and this study.**
(DOCX)
