# Supplementary material for: Differential Gene Expression between African American and European American Colorectal Cancer Patients
Source: PLoS One. 2012 Jan 19;7(1):e30168. doi: 10.1371/journal.pone.0030168 (PMC3261881; doi:10.1371/journal.pone.0030168)
Supplement: Table S1 — Ingenuity Pathway Analysis of association of differentially expressed genes with top bio functions. (DOCX) [file pone.0030168.s001.docx]

| ***Disease and Disorders*** |  |  |
| --- | --- | --- |
| **Name** | **p-value** | **# Molecules** |
| Inflammatory Response | 1.66E-05 - 4.82E-02 | 6 |
| Hepatic System Disease | 8.91E-04 - 4.97E-02 | 3 |
| Developmental Disorder | 1.92E-03 - 4.82E-02 | 4 |
| Genetic Disorder | 1.92E-03 - 4.82E-02 | 11 |
| Neurological Disease | 1.92E-03 - 4.21E-02 | 10 |
