# Supplementary material for: Differential Gene Expression between African American and European American Colorectal Cancer Patients
Source: PLoS One. 2012 Jan 19;7(1):e30168. doi: 10.1371/journal.pone.0030168 (PMC3261881; doi:10.1371/journal.pone.0030168)
Supplement: Table S2 — Comparison of top 40 differently expressed genes in five other CRC microarray studies and this study. (DOCX) [file pone.0030168.s002.docx]

| *Top-20 Over-expressed Genes Across 5 Colorectal Cancer Studies* | | | *Over-expressed genes in this study* |
| --- | --- | --- | --- |
| Median Rank | **p-Value** | **Gene** |  |
| 14.00 | 2.59E-08 | **NME1** | x |
| 23.00 | 8.09E-08 | **HSP90AB1** |  |
| 29.00 | 5.26E-05 | **S100A11** | x |
| 31.00 | 8.65E-17 | **MMP7** | x |
| 34.00 | 1.67E-16 | **TH1L** |  |
| 48.00 | 5.43E-10 | **RPN2** | x |
| 52.00 | 1.84E-06 | **HMGA1** |  |
| 56.00 | 7.87E-18 | **HMGB1** | x |
| 61.00 | 1.02E-17 | **CKS2** | x |
| 64.00 | 1.40E-17 | **HEATR2** | x |
| 74.00 | 4.72E-04 | **CCT3** | x |
| 76.00 | 2.89E-04 | **POLR1D** | x |
| 81.00 | 1.05E-05 | **CDK4** | x |
| 92.00 | 7.31E-14 | **CD44** |  |
| 96.00 | 2.59E-05 | **CDH3** | x |
| 98.00 | 1.89E-16 | **NOB1** | x |
| 106.00 | 2.88E-05 | **EIF2S2** | x |
| 109.00 | 3.71E-10 | **IFT52** |  |
| 117.00 | 5.25E-16 | **SULF1** | x |
| 117.00 | 5.60E-05 | **COL11A1** | x |

| *Top-20 Under-expressed Genes Across 5 Colorectal Cancer Studies* | | | *Under-expressed genes in this study* |
| --- | --- | --- | --- |
| Median Rank | **p-Value** | **Gene** |  |
| 17.00 | 2.80E-18 | **SRPX** | x |
| 20.00 | 3.18E-08 | **ADH1B** | x |
| 27.00 | 1.25E-11 | **CDH19** | x |
| 39.00 | 5.43E-14 | **AGXT2L2** | x |
| 54.00 | 4.62E-13 | **MT1B** | x |
| 64.00 | 1.04E-05 | **MT1E** | x |
| 68.00 | 4.39E-14 | **NR3C2** | x |
| 72.00 | 6.90E-14 | **SLC4A4** | x |
| 76.00 | 3.16E-06 | **ADH1C** | x |
| 81.00 | 3.72E-06 | **DPT** | x |
| 82.00 | 1.78E-04 | **GPD1L** |  |
| 96.00 | 1.01E-11 | **LY9** |  |
| 98.00 | 9.23E-13 | **MT1H** | x |
| 101.00 | 1.35E-11 | **PDE2A** | x |
| 101.00 | 5.60E-11 | **SETBP1** | x |
| 106.00 | 2.00E-03 | **TSPAN7** | x |
| 107.00 | 2.54E-05 | **PCK1** | x |
| 111.00 | 3.19E-12 | **ATP9B** |  |
| 112.00 | 3.03E-05 | **MAOB** | x |
| 113.00 | 3.57E-12 | **CDKN1A** | x |
